# Effects of Altered Haptic Feedback Gain Upon Balance Are Explained by Sensory Conflict Estimation

**DOI:** 10.1111/ejn.16670

**Published:** 2025-01-14

**Authors:** Raymond F. Reynolds, Craig P. Smith, Lorenz Assländer

**Affiliations:** ^1^ School of Sport, Exercise & Rehabilitation Sciences University of Birmingham Birmingham UK; ^2^ Human Performance Research Centre University of Konstanz Constance Germany

**Keywords:** balance, postural stability, sensorimotor integration, sensory system

## Abstract

Lightly touching a solid object reduces postural sway. Here, we determine the effect of artificially modifying haptic feedback for balance. Participants stood with their eyes closed, lightly gripping a manipulandum that moved synchronously with body sway to systematically enhance or attenuate feedback gain between +2 and −2, corresponding to motion in the same or opposite direction to the body, respectively. This intervention had a systematic effect on postural sway, which exhibited an asymmetric u‐shape function with respect to haptic feedback gain. Sway was minimal around zero gain, corresponding to a static object. Sway increased slightly at gains below −0.25 but increased greatly at gains above +0.25. At +2, it was approximately double that of a no‐touch condition. Mean interaction force between the hand and manipulandum remained < 0.9 N throughout, although it increased slightly at extreme gains. Cross‐correlations between hand force and trunk position were highest during conditions of least sway, suggesting that higher quality haptic feedback is associated with greater sway reduction. We successfully replicated the sway behaviour using a feedback control model that attenuated haptic feedback signals when the discrepancy between haptic and proprioceptive signals reached a threshold. Our findings suggests the CNS can utilise augmented haptic feedback for balance, but only with relatively small changes to natural feedback gain. In healthy volunteers, it offers minimal benefit over a static object. Haptic feedback is therefore optimal when motion is physiologically realistic and subtle enough to be misinterpreted as self‐motion.

## Graphical abstract

Light touch improves balance. Here, we systematically manipulated haptic feedback gain by asking participants to lightly grip a robotic manipulandum, which moved synchronously with body sway. Sway was lowest when the manipulandum was stationary or moved slightly in the opposite direction (−ve gain). At gain +2, this feedback signal was actively destabilising. We successfully recreated the behaviour using a model that attenuated haptic feedback signals when the disagreement between proprioception and haptics exceeded a certain threshold.

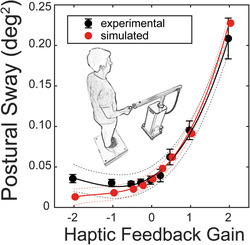

**Abbreviations**
CNS: central nervous system
COM: centre of mass
ANOVA: analysis of variance
PSD: power spectral density
SEM: standard error of the mean

## 1 Introduction

When standing, the central nervous system (CNS) makes use of a wide range of sensory cues to estimate body sway. This includes visual, vestibular and proprioceptive feedback. It can also include haptic feedback when touching an earth‐fixed object (Holden, Ventura, and Lackner 1994; Smith et al. 2017). Touch reduces spontaneous sway by ~25%, which is similar in magnitude to the effect of vision (Jeka and Lackner 1995; Jeka 1997). This occurs even when contact forces are too low to offer significant mechanical support (i.e., < 1 N). Exceeding this force provides minimal additional benefit. Hence, the benefits of touch are achieved mainly through enhanced sensory feedback of body sway (Kouzaki and Masani 2008). This feedback could originate from at least two sources. Firstly, unpredictable body movement will alter the force experienced at the fingertip, as registered by cutaneous afferents. Secondly, relative motion between the hand and body occurs during postural sway. The concomitant movement of the wrist, elbow and shoulder joints will be signalled by muscle spindles and/or Golgi tendon organs.

Although contact with static objects reduces spontaneous sway, moving haptic stimuli *evoke* body sway (Jeka et al. 1997; Oie, Kiemel, and Jeka 2001; Reynolds and Osler 2014; Assländer, Smith, and Reynolds 2018; Michel et al. 2023). Touching a slowly oscillating object induces sway in the same direction and frequency as the stimulus, suggesting the CNS misinterprets the object motion as self‐motion and attempts to align the body with the moving reference (Vérité et al. 2020). In general, responses to sensory perturbations attenuate steeply beyond ~0.5–1 Hz (Jeka et al. 1997; Peterka 2002; Dakin et al. 2007). This suggests that in order to evoke sway, stimulus motion must be slow/subtle enough to be misconstrued as self‐motion rather than object motion. Indeed, slow‐moving haptic stimuli have previously been utilised to directly control human balance. Verite, Bachta, and Morel (2014) demonstrated that body position can be unconsciously driven to a predetermined location by a haptic stimulus. This raises the possibility that balance could be enhanced by haptic technology.

For a moving haptic stimulus to provide useful information for balance, it should be tailored in a manner to give feedback of ongoing sway, which is precisely what we attempt here. We provide haptic input to standing participants using a robotic arm controlled in real time by postural sway. The endpoint of the robot arm is gripped lightly between finger and thumb while standing quietly with the eyes closed. This setup allows us to manipulate the gain between body and robot movement. For example, the robot can be made to move in the same or opposite direction as body sway, corresponding to positive and negative feedback gain, respectively.

With this approach, we ask the following questions. Firstly, can balance be enhanced by artificially altering haptic feedback gain? Increasing the relative motion between hand and body for any given amount of body sway should enhance sensory detection of sway and therefore improve postural control. Secondly, can the CNS utilise *reversed* haptic feedback gain? Normally, when grasping a stationary object, rightward body sway corresponds to leftward relative motion of the hand with respect to the body. But what if this normal pattern of motion is artificially reversed? Moving the robot in the same direction as body motion, in this way, would provide non‐physiological feedback compared to the ‘natural’ sensations afforded by a static object. Research using vibrating tactors applied to the torso suggests that non‐physiological tactile feedback can be successfully used by the CNS for balance (Wall et al. 2001; Schulleri et al. 2024). Any haptic feedback gain ≠1 (a gain of 1 being equivalent to holding a free‐floating object), including reversed feedback, could therefore theoretically provide body sway information to the CNS. Hence, reversed haptic feedback could augment balance despite being ‘unnatural’. Third, is sway reduction directly related to quality of haptic feedback obtained at the hand? Specifically, is sway control directly connected to the precision with which hand force reflects body sway? We address this question by analysing the correlation between hand force and body sway across the different gain conditions. Lastly, what are the neural mechanisms by which haptic feedback is processed for balance control? To answer this question, we use a modified version of our feedback control model of standing. This model previously explained the sway response to external perturbations of a haptic reference (Assländer, Smith, and Reynolds 2018). The model has three main components: (1) a space‐referenced feedback loop, generating torque relative to body orientation in space; (2) a touch‐referenced feedback loop, stabilising the body relative to the haptic reference; and (3) estimation of haptic stimulus motion in space based upon a velocity threshold. This latter component is crucial for disambiguating self versus externally imposed haptic motion. Here, we apply these principles to explain the effects of altered haptic gain on postural sway.

## 2 Methods

### 2.1 Participants

Fourteen participants gave written informed consent to participate in the experiment (mean age: 23 ± 6.9 years; seven females). Ethical approval was received from the School of Sport, Exercise and Rehabilitation Sciences at the University of Birmingham (CM 191017‐1). All volunteers gave written informed consent, and the experiment was conducted in accordance with the Declaration of Helsinki.

### 2.2 Apparatus and Protocol

**FIGURE 1:**
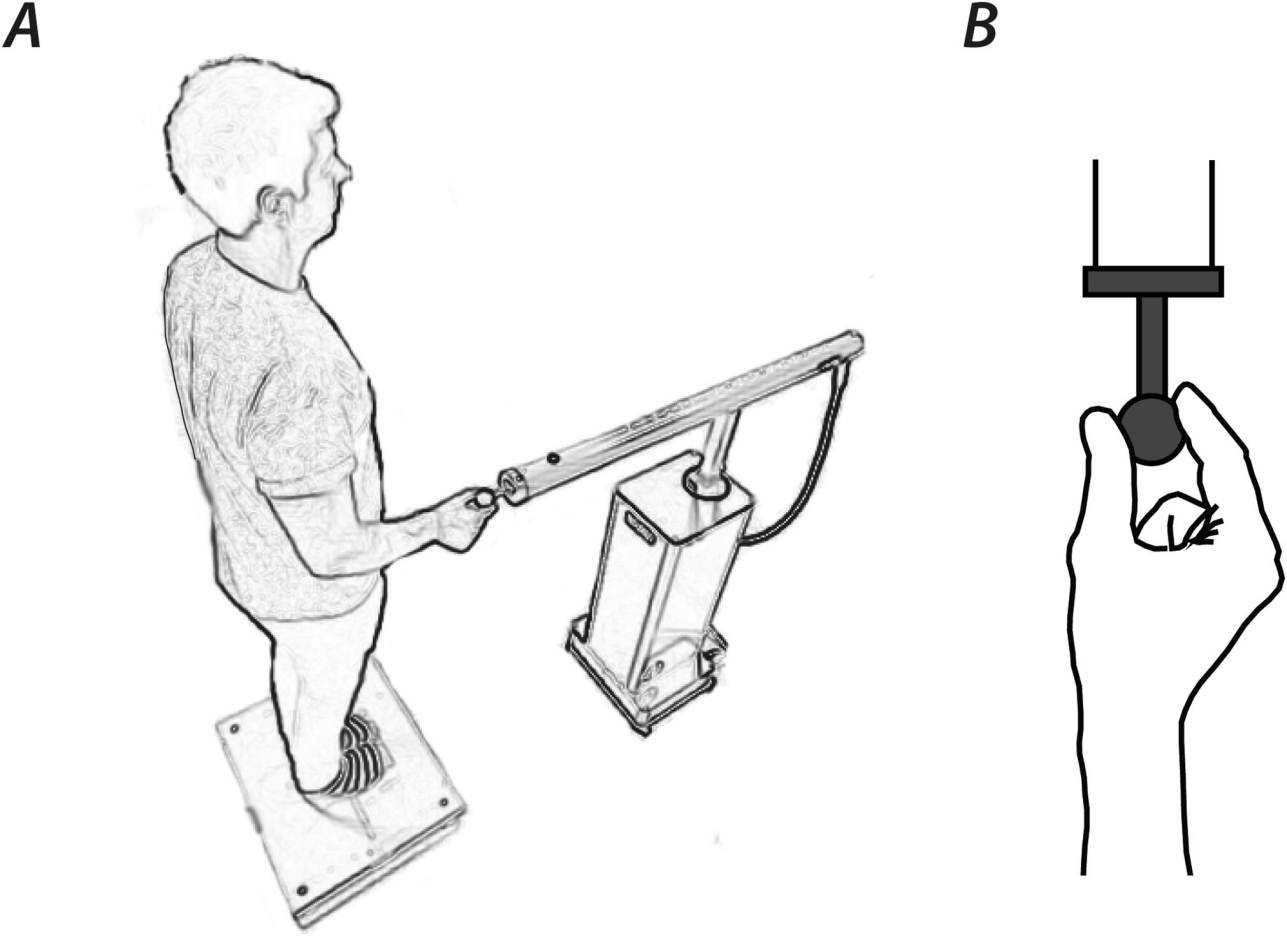
Experimental setup. (A) Participants stood on a force platform while lightly gripping the manipulandum of the MOOG haptic master. Interaction forces between the person and haptic master were transduced by a triaxial force sensor embedded within the handle of the manipulandum. (B) The hand grip posture in detail from above. Participants were instructed to maintain a light grip throughout.

Participants stood unshod, wearing socks with feet together and eyes closed, lightly gripping the end of a robotic arm with their right index finger and thumb (see Figure 1; HapticMaster, Moog FCS, Netherlands; Van der Linde et al. 2002). They were instructed to grip the ball of the manipulandum lightly between finger and thumb using the hand posture depicted in Figure 1B. Participants were aware that the manipulandum might move but were not given explicit information about the nature of the movement. They were simply instructed to maintain a light grip throughout while standing still but relaxed. The robot arm endpoint was located approximately 400 mm anterior to the ankle joint, 200 mm lateral to the body midline, at a height of 1100 mm. Embedded within the handle of the robot arm was a triaxial force sensor. This sensor did not measure grip force but allowed manual interaction forces between the person and manipulandum to be measured. The device was controlled in the medio‐lateral and anterior–posterior axes using Simulink Desktop Real‐Time software (Mathworks, USA). A Fastrak motion tracking device (Polhemus, USA) was used to measure body sway and was attached to the person's back at the same height as the touch reference. The motion tracking signal was acquired at 120 Hz through the serial port into Simulink where it was used as the control signal for the robot. We determined the lag between the motion tracker and manipulandum position to be approximately 60 ms, from the peak cross‐correlation between the two signals, without considering the closed‐loop dynamics. The gain between body sway and robot motion was systematically modulated to provide the following nine gain values: −2, −1, −0.5, −0.25, 0, 0.25, 0.5, 1 and 2. A gain of 0 indicates that the robot was static. Positive gain means that the robot moves in the same direction as body sway. With negative gain, it is reversed. This applies to motion in both axes. For example, with a gain of −2, if the person sways left by 10 mm and backward by 15 mm, the robot will simultaneously move right by 20 mm and forward by 30 mm. A gain of 1 indicates that the robot was commanded to move with the body, such that relative motion between hand and body was minimised. Postural sway was studied in trials of 50 s duration. The first 10 s of each trial was discarded from analysis to allow for any settling in to each gain setting. Each gain setting was repeated five times, leading to a total of 50 trials. Trial order was randomised. Although they were aware that the manipulandum might move, participants were not made aware which haptic gain condition they were about to experience prior to each trial.

**TABLE 1:** Model parameters.

Body properties	COM height *h* (m)	Body mass *m* (kg)	Inertia *J* (kg m^2^/s^2^)		
	0.96	68.5	76.4		

Balance control	Proportional loop gain *Kp*	Derivative loop gain *Kd*	Force feedback loop gain *Glp*	Time delay *dt* (s)	Force feedback filter constant *Flp*
	1.19	0.30	0.0021	0.106	20

Light touch feedback	Loop gain *LT*	Estimator gain *Ge*			
	0.29	0.58			

*Note:* These parameters were fixed according to Assländer, Smith, and Reynolds (2018).

### 2.3 Postural Control Model

**FIGURE 2:**
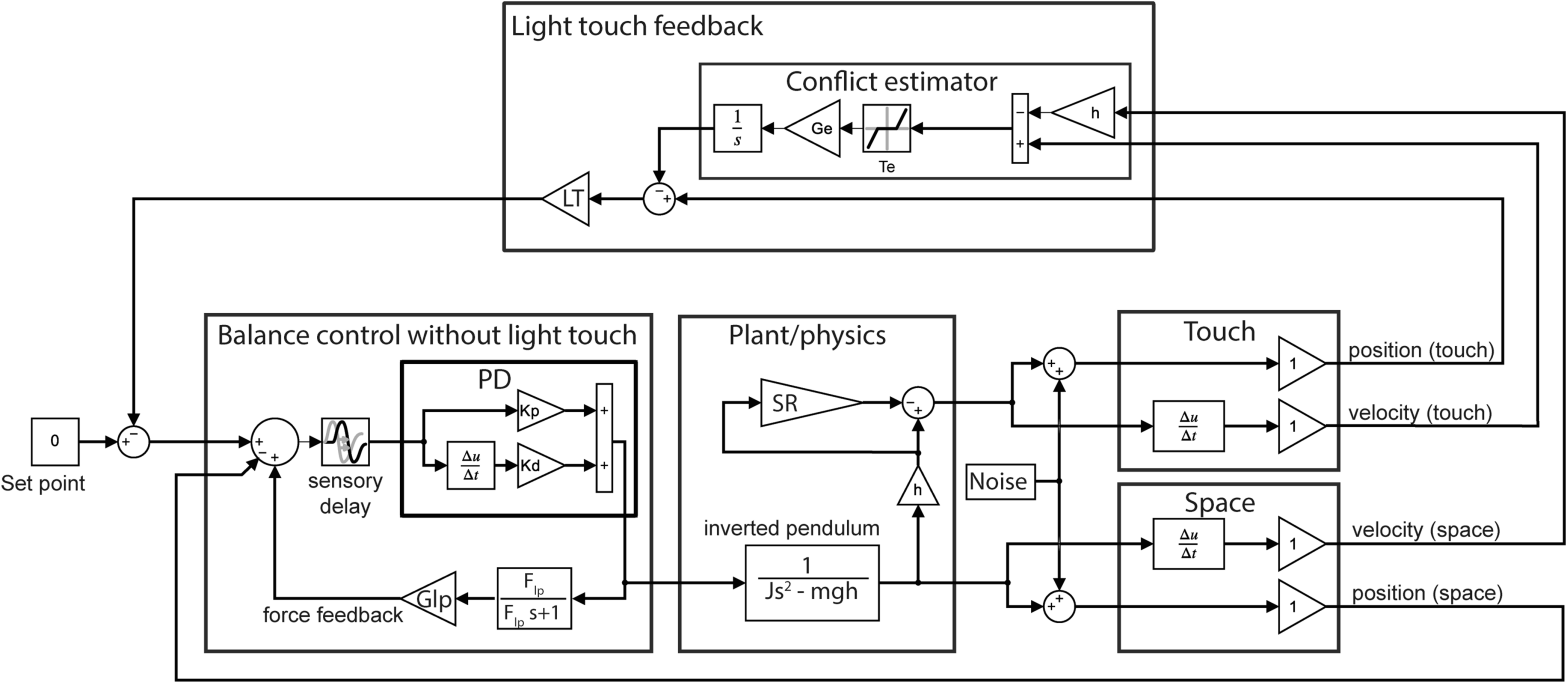
Modelling light touch feedback for balance control. The body is represented by an inverted pendulum. Two sensory feedback channels convey light touch and all other sensory information (‘space’) to a proportional‐derivative controller, with a lumped delay accounting for neural conduction, muscle activation dynamics, etc. The conflict estimator calculates the difference between the two channels. If this difference exceeds a threshold, the light touch information is subtracted from the feedback loop. Sway is driven by a single noise source added to both sensory channels. The noise gain and filter constant and the conflict threshold (Te) were allowed to vary. All other parameters were fixed (see Table 1).

We used Simulink to create a feedback control model to simulate and explain the pattern of sway behaviour under different haptic feedback gains (Figure 2). This is a modified version of a previously published model in which the body is represented by a linearised inverted pendulum (Assländer, Smith, and Reynolds 2018). As the pendulum moves, sensory feedback is relayed back to a proportional‐derivative controller with a delay. The controller then generates a corrective torque signal to restore pendulum position to the set point. We incorporated two separate sensory systems, one for light touch cues and another carrying all other sway‐relevant information encoding body position in space, including vestibular and proprioceptive reference to the floor. For simplicity, we refer to these two sources of sensory information as ‘touch’ and ‘space’. In the model, both sensory systems are assumed to have ideal transfer characteristics and are available as both position and velocity cues. The touch feedback signal consists of the horizontal distance between the hand and the body. The space feedback signal consists of body angular orientation relative to vertical. Crucially, the model includes an additional loop, termed the ‘conflict estimator’. This is designed to disambiguate hand‐on‐body motion due to self‐motion (i.e., body sway) from that due to object motion (i.e., robot motion). The difference in body angular velocity estimated from touch and space feedback loops is calculated. When this difference exceeds a specific threshold, the value of the difference is subtracted from the touch feedback loop. In this way, the model attenuates touch feedback caused by object motion larger than the threshold velocity. Notably, the compensation is only effective if the estimated touch motion exceeds this threshold. This mechanism was shown in the previous study to explain sensory reweighting when the amplitude of a moving haptic stimulus was changed (Assländer, Smith, and Reynolds 2018). In that previous study, the model simulation was driven by a fixed perturbation signal that matched the empirical haptic perturbation signal, and experimental sway variability was reduced by averaging. In other words, the model mimicked sway responses to external stimuli assuming noise‐free sensory and motor signals. As there is no fixed external stimulus here, we instead drive pendulum sway by applying random noise, that is, we implement noise sources to mimic the characteristics of the internally driven random sway component, instead of a sway response to an external stimulus. The noise source was added to both sensory inputs (see Figure 2). Noise sources were low‐pass filtered and gain scaled, where both the filter constant and the noise gain were subject to a parameter optimisation process. Several noise positions and using two noise inputs at different locations were tested, and they all exhibited similar or worse performance. All other parameters were fixed to the values identified in the previous study (Assländer, Smith, and Reynolds 2018), because the model dynamics cannot be estimated without a broad characterisation of the system using external stimuli (Pintelon and Schoukens 2004). One important exception was the threshold, which was included as a free parameter in the optimisation process, as non‐linear systems interact with noise through processes such as stochastic resonance.

#### 2.3.1 Simulations and Optimisation Procedure

The goal of the optimisation was to estimate the noise characteristics that best reproduce the experimental sway response characteristics using the model described above. The simulations were designed analogous to the experiments. For each iteration of the optimisation approach, the model ran for 2810 s, where the first 10 s was discarded and the rest were cut into 40‐s pieces, from which the mean spectral characteristics were calculated analogous to the experimental data analysis (see below). The objective function was based on mean velocity power spectra from 0.025 to 4 Hz. The objective was the squared difference of experimental and simulation spectra, weighted by 1/f and summed across all frequencies and across all sway reference gain conditions. The three parameters (gain and filter constant of the noise source and the threshold level) were fitted using ‘lsqnonlin’ from the Matlab Optimization toolbox. We used repeatable random inputs during the optimisation to ensure convergence. To test the robustness with respect to different noise realisations, the optimisation was run 20 times with different noise inputs. Finally, the optimisation was manually started from several initial parameter values for one noise realisation to avoid local minima. The algorithm always converged to the same parameter values.

### 2.4 Data Analysis and Statistics

Body sway was measured as the integrated value of the power spectral density (PSD) of trunk position and velocity in the anterior–posterior plane, calculated between 0.025 and 4 Hz. Velocity was first calculated by differentiating the trunk position signal after applying a second‐order 5‐Hz low‐pass Butterworth filter. Hand force was measured by calculating the magnitude of the force vector derived from the triaxial force sensor embedded within the handle of the manipulandum: $\left|F\right|=\sqrt{Fx^{2}+Fy^{2}+{\mathrm{Fz}}^{2}}$. This provided a measure of the absolute force between the person and manipulandum irrespective of direction. The mean value over the duration of each trial was taken for each condition.

Cross‐correlations between trunk position and hand force in the anterior–posterior plane were calculated using the ‘xcorr’ function in Matlab. These were unbiased and normalised, producing a correlation (*R*) value between +1 and −1 for each time lag. Magnitudes and time lags of peak *R* values were measured for subsequent statistical analysis.

The effects of haptic feedback gain upon integrated values of trunk position and velocity spectra, hand force and cross‐correlation values were determined by repeated‐measures ANOVA (Jamovi Version 2.3). Following a significant ANOVA result, pairwise comparisons were performed using the Tukey correction. *p* < 0.05 was considered significant for all tests.

## 3 Results

### 3.1 Manipulating Haptic Feedback Gain

**FIGURE 3:**
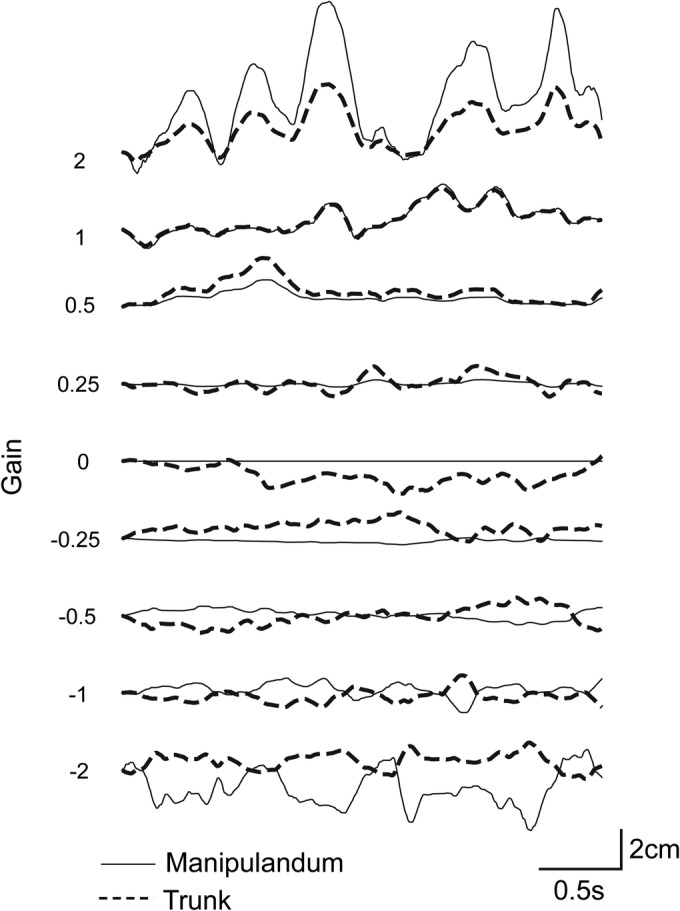
Manipulating haptic feedback gain during standing. Sagittal trunk position from a single representative participant is shown alongside position of the robotic manipulandum. The robot was controlled in real time by the trunk position marker at nine different levels of feedback gain. Motion was simultaneously controlled in the frontal plane in the same way (not shown).

We attempted to manipulate the movement of a robotic manipulandum that participants lightly gripped such that we could systematically alter the magnitude of haptic feedback signals for balance. Figure 3 depicts motion of the body and robotic manipulandum in the sagittal plane for a representative participant during all nine conditions of altered gain. The pattern of manipulandum movement is clearly time‐locked to trunk position but the direction and amplitude of this motion changes according to gain condition. At gain = 0, the manipulandum is stationary with respect to external space. In this case, ongoing body sway occurs, but the position of the hand remains locked to the stationary manipulandum. During positive gain conditions, the robot moves in the same direction as the body. For example, at gain = +2, as the participant sways forward by 2 cm, the manipulandum moves in the same direction by 4 cm. In contrast, at negative gains, it moves in the opposite direction. At gain = +1, the manipulandum is made to move in the same direction and with the same amplitude as the body. This mimics a free‐floating object, providing minimal feedback of body sway.

### 3.2 Body Sway

**FIGURE 4:**
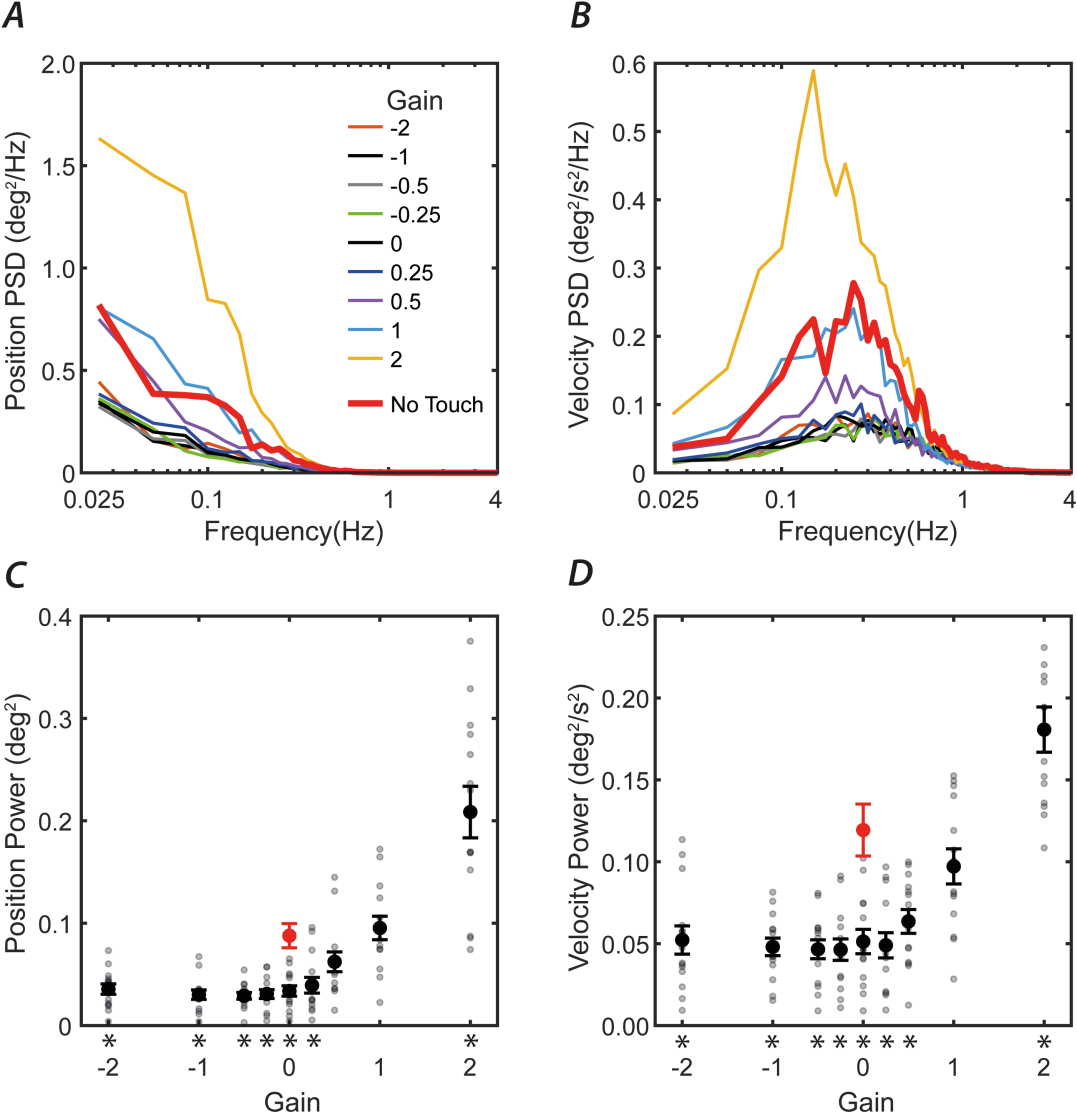
Body sway under different haptic feedback gains. Group mean power spectral density of angular body position (A) and velocity (B) in the sagittal plane is shown for all gain conditions. (C,D) Integrated values of power spectra. Individual values are depicted by small grey dots; mean values are depicted by heavy circles. Red dots depict the no‐touch condition. Bars show one standard error of the mean. Asterisks depict significant difference from the no‐touch condition (*p* < 0.05, Tukey post hoc comparison).

Figure 4A depicts PSD of trunk angular position in the sagittal plane. For all gain conditions, position PSD is maximal at the lowest frequency and steeply declines as frequency increases. Integrated PSD position values are shown in Figures 4C. There is a significant effect of tactile gain upon trunk position (*F*_9,117_ = 41.5; *p* < 0.001). Integrated position power exhibits an asymmetric function with respect to gain, being minimal between −1 and 0 gain, increasing very slightly as gain reduces to −2 and increasing by a much greater amount as it increases to +2. When compared to the no‐touch condition (red dot in Figure 4C), all gains between −2 and +0.25 cause a significant reduction in position PSD values, whereas gain +2 is associated with in a large increase (*p* ≤ 0.014; Tukey‐corrected post hoc comparisons; see asterisks in Figure 4C). When compared to the zero gain condition (equivalent to a static object), sway is significantly greater during both +1 and +2 gains (*p* ≤ 0.002). However, no conditions exhibit a significant reduction in position PSD compared to the zero gain condition (*p* ≥ 0.09).

Figure 4B depicts trunk angular velocity PSD in the sagittal plane. Velocity PSD forms a peak around 0.1–0.3 Hz for all conditions. Integrated PSD velocity values are shown in Figures 4D. There is a significant effect of tactile gain condition upon trunk velocity (*F*_9,117_ = 52.1; *p* < 0.001). All gains between −2 and +0.5 result in significantly lower sway compared to the no‐touch condition (*p* ≤ 0.007). At gain +1, it is not different (*p* = 0.47), whereas at +2 sway is greater than the no‐touch condition (*p* = 0.008). When compared to the zero gain condition, the situation for velocity is the same as for position. That is, sway is significantly greater than the zero gain condition for both +1 and +2 gains (*p* ≤ 0.002), but no conditions exhibit a reduction in velocity PSD compared to the zero gain condition (*p* ≥ 0.36).

### 3.3 Hand Force

**FIGURE 5:**
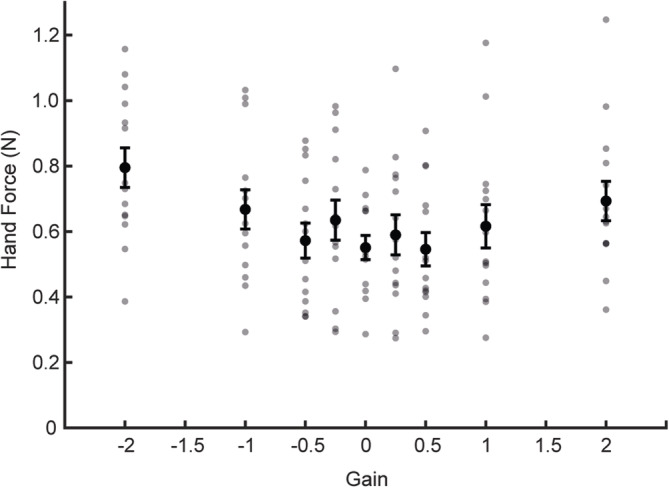
Hand force during light touch contact. Solid black circles depict group mean of the absolute magnitude of the 3D force vector, as detected by the triaxial force transducer embedded within the handle of the robotic manipulandum. Grey dots show individual participants. Bars show 1 standard error of the mean.

Figure 5 depicts the mean value of the absolute force between the hand and the robotic manipulandum, calculated as the absolute magnitude of the 3D force vector. Mean force remained less than 0.9 N for all gain conditions, with slightly greater force at the most extreme gains (Figure 5; *F*_8,104_ = 9.8; *p* < 0.001).

### 3.4 Comparison of Empirical and Model Data

**FIGURE 6:**
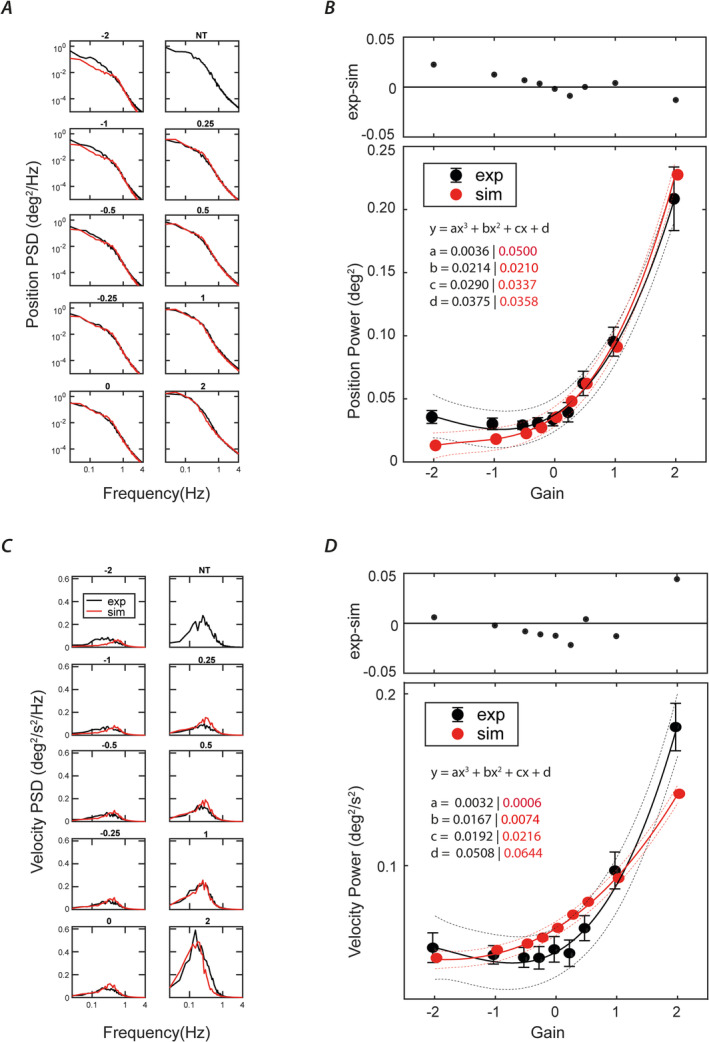
Comparison of model and experimental data. (A) Power spectral density for sagittal body angular position. (B) Lower graph shows integrated power; upper graph shows the difference between experimental and simulated data. (C,D) The same for body angular velocity. Solid lines in (B) and (D) represent third‐order polynomial fits. Dashed lines show 95% confidence limits of the fitted lines. Embedded values represent the parameters for the fitted equation. Experimental and modelled data are shown in black and red, respectively, for all gains.

Figure 6 shows power spectra for simulation data alongside empirical data across all haptic gain conditions. The simulated spectra closely resemble both the amplitude and shape of the empirical spectra for both sway position and velocity (Figure 6A,C, respectively). Integrated PSD values for both position and velocity are maximal at gain = +2, with a steep fall as gain approaches 0 (red circles in Figure 6B,D, respectively). As gain becomes negative, there is a further shallow reduction in both. This contrasts with the empirical data, where sway increases slightly over the same gain values. The differences between experimental and simulated data can be seen in upper graphs of Figure 6B,D.

### 3.5 Relationship Between Hand Force and Body Position

**FIGURE 7:**
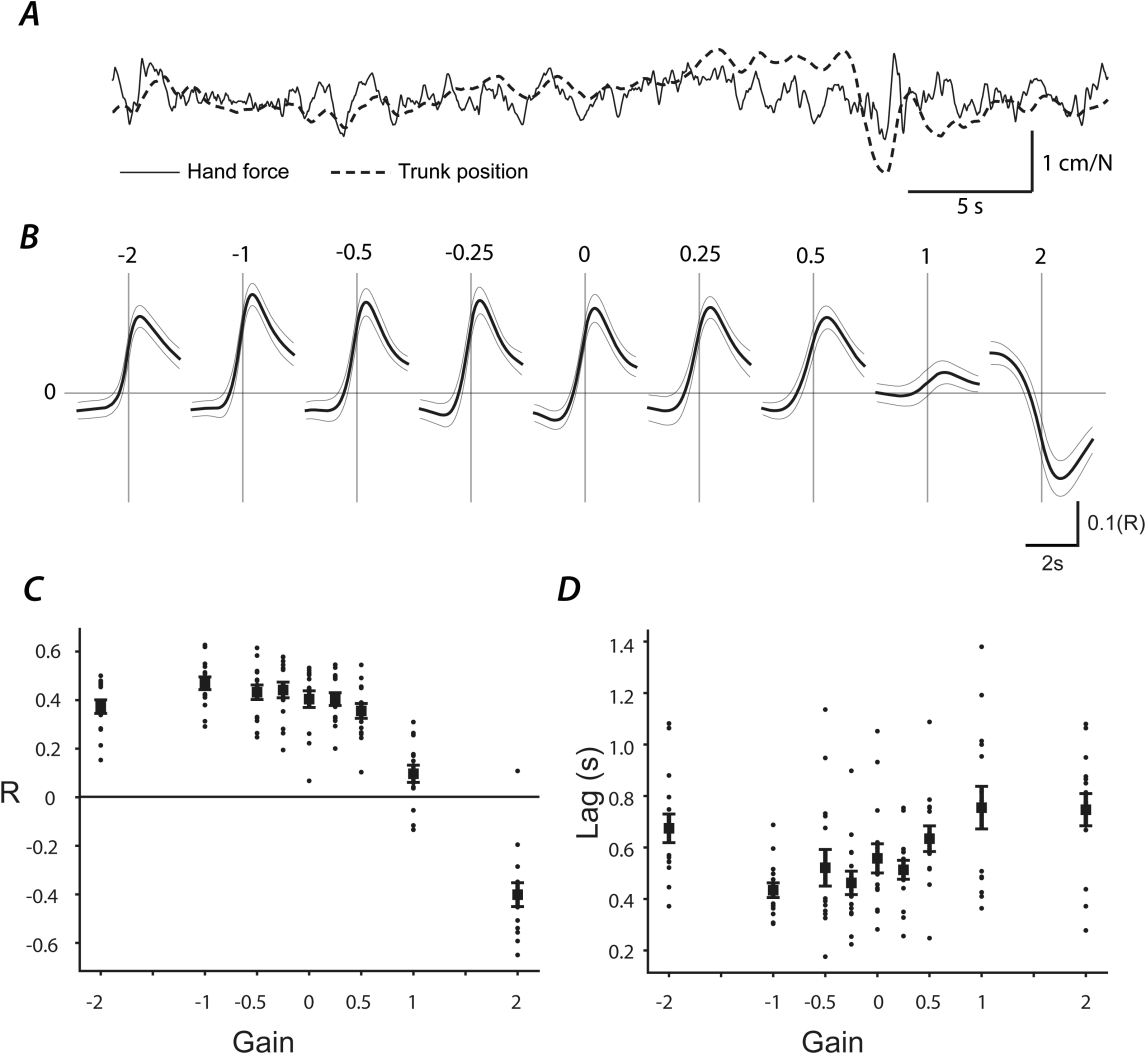
Cross‐correlation of trunk position and hand force. (A) Representative examples of sagittal trunk position and hand force are shown for the −0.25 gain condition. Positively directed values correspond to forward force acting on the robot and forward body sway. (B) Trunk position–hand force cross‐correlations for each gain condition. Mean +/− 95% confidence intervals are shown. Horizontal and vertical lines depict zero correlation and lag, respectively. (C,D) Group mean peak correlation coefficients and lags (+/−1 SEM). Mean data are shown by solid squares, whereas individual subject data are depicted by black dots.

To gain further insight into the nature of the haptic sensory input, we analysed the relationship between body position and hand force. Figure 7A shows the representative examples of these signals for the −0.25 gain condition. The hand force signal appears to contain higher frequency components than body position. However, there is a general concordance between the two time series, forward body sway being associated with an increase in forward‐directed force acting on the robot arm. Cross‐correlations between the two signals reveal a pronounced peak for gains < 1 (Figure 7B). This is attenuated for gain = +1 condition and reversed for the +2 condition. Overall, peak correlation values (Figure 7C) precisely mirror body sway values in Figure 4C,D, being maximal between gains of 0 and −1, minimal at gain = 1 and negative for gain = +2 (main effect of gain upon *R* value: [*F*_8_ = 96, *p* < 0.001]). Peak correlations always occurred at positive time lags, indicating that body position preceded hand force by 0.4–0.7 s (main effect of gain upon lag: [*F*_8_ = 12.5, *p* < 0.001]).

## 4 Discussion

We manipulated haptic feedback gain while standing participants lightly grasped a robotic arm with their eyes closed. In keeping with previous research, when the robot arm was stationary (gain = 0), postural sway was significantly reduced by 55%–62% compared to no contact. When the robot moved synchronously in the opposite direction to body motion (i.e., any gain below zero), sway remained low, being minimal between gains 0 and −1. However, when the robot moved in the *same* direction as the body, haptic feedback was less useful. At gain +2, it was actively detrimental, with sway being approximately double that of the no‐contact condition (Figure 4C,D). This overall pattern of behaviour was replicated by a feedback control model that attenuated the touch feedback signal when the disagreement between touch and space feedback of body sway reaches a certain threshold.

The first question we posed was whether balance could be enhanced by altering haptic feedback gain. To answer this, we assume that postural sway is a proxy for balance control, with less sway corresponding to better control. Compared to the no‐touch condition, sway was indeed reduced by haptic feedback for all conditions except +1 and +2 gains. At +1 gain, the robotic manipulandum should essentially act as a free‐floating object, and sway was not significantly different from no touch in this condition. This confirms the robot performed sufficiently fast to track body motion so as not to provide any significant sway feedback. Between −2 and +0.5 gains, feedback was successfully utilised by the CNS to reduce sway. As contact forces were < 0.9 N, we can be confident that these reductions were primarily driven by sensory input rather than mechanical support. But despite minimum sway values occurring around gains of −0.25 and −0.5, they were not statistically distinguishable from those observed during gain 0. Hence, the artificial feedback we supplied did not improve postural control beyond that afforded by a static object. The participants we studied were young healthy volunteers who can be expected to have optimal sensorimotor control. Thus, it may be the case that balance performance was near ceiling, with little room for improvement. In contrast, older or neurologically impaired individuals might exhibit greater benefit from altered haptic feedback gains < 0. Afzal et al. (2015) provided haptic balance cues in the form of force feedback of body tilt using a Phantom Omni device. They found that force feedback significantly reduced postural sway compared to no feedback at all. However, it is not clear whether they define their ‘no‐feedback’ condition as a stationary Omni device or no contact at all. If it is the former, then they observed an impressive reduction in sway compared to a static object. However, they studied people who were inherently unstable, either because they were standing on one leg or in Romberg stance (both with eyes closed) or because they had a stroke. Despite methodological differences and the lack of clarity regarding the no‐feedback condition, their study does raise the possibility that real‐time haptic feedback may confer the most benefit when baseline sway is high due to pathology or difficult stance conditions.

A related question was whether *reversed* haptic feedback can be successfully utilised by the CNS. At any gain greater than +1, the relative motion between hand and body is effectively reversed when compared to touching a static object. At a gain of +2, the amplitude of relative hand–body motion is identical to that of the gain 0 condition, but in the opposite direction. Both conditions theoretically provide the same *quantity* of haptic feedback, but reversed. Indeed, the maximum absolute cross‐correlation values between hand force and body sway were very similar for both conditions (*r* value ~0.4), suggesting the level of haptic information was similar. Nevertheless, during the gain +2, postural sway was considerably greater than no touch, by over 100% when measured as sway position spectral power. Hence, haptic feedback was actively destabilising in this case. Previous research suggests that haptic information can be useful for balance even when delivered in a manner which deviates from ‘natural’ physiological feedback. Wall et al. (2001) applied 250‐Hz vibration pulses to the shoulder in a manner that encoded head tilt during standing. They observed a ~33% reduction in sway compared to no tactile feedback. Although the precise nature of their vibrational feedback might be considered unphysiological, it did contain concordant directional information; rightward sway resulted in stimulation of the right shoulder. Our results suggests that the CNS cannot utilise reversed feedback. Whether this is a ‘hard‐wired’ property of the CNS, or is amenable to adaptation, is open to question.

By calculating the cross‐correlation between hand force and body position, we sought to assess the quality of haptic feedback during each gain condition. A high correlation value implies that force imparted to the hand contains higher quality body position information. A lower value implies the force is contaminated with noise or information unrelated to sway. For all conditions except +1 and +2, correlation values were approximately 0.4, meaning that a higher proportion of the force time series contained useful body position feedback. It is noteworthy that the pattern of correlation values closely mirrors that of postural sway. As sway increases, correlation values tend to decrease. This association suggests that the ability to reduce sway is closely related to the quality of cutaneous force feedback at the level of the hand. Of course, this does not exclude the role of other receptors, which will also signal changes in relative hand–body motion, such as muscle spindles and Golgi tendon organs. The cross‐correlations also exhibit considerable lag between body position and hand force, around 400–700 ms. This corroborates the notion of a sensorimotor process, rather than mechanical support.

Finally, a key component of the current study was our attempt to model the underlying mechanisms. We adapted the feedback control model of Assländer, Smith, and Reynolds (2018) to include a conflict estimator. Just like vision, haptic feedback can be inherently ambiguous. The CNS must continuously determine whether sensory feedback is caused by self‐motion (reafference) or external motion (exafference). The model achieved this by performing a continuous comparison of body velocity estimated by touch versus that estimated by all other sensory inputs combined (‘space’). Once the difference exceeded a certain threshold, touch information was attenuated, effectively reweighting to space information. The threshold was left as a free parameter for the model to tune. Incorporating this conflict estimator allowed the model to recreate the characteristics of the empirically observed sway data. The spectral sway characteristics were well matched for both position and velocity (Figure 6A,C). The overall effect of haptic feedback gain condition upon sway was also broadly recreated, particularly the asymmetry between positive and negative gains (Figure 6B,D). Specifically, negative gains were most beneficial for sway, whereas positive gain was ultimately destabilising. As we observed in the empirical data, the +2 gain was by far the most unstable. It is worth noting that the model conflict estimation is a continuous process. Thus, despite the +2 condition presumably resulting in considerable exclusion of haptic feedback, there were nevertheless times when the conflict was below threshold and the feedback was adhered to, despite being destabilising. Overall, the model performance suggests that a sensory conflict estimator may be a key component of CNS postural control, allowing haptic information to be effectively ‘tuned’ in or out as it becomes more or less reliable.

However, there was a notable discrepancy between the model and empirical data. As gain went below zero, model sway continued to reduce in amplitude. This contrasts with the empirical sway data that plateaued and slightly increased as gain went from 0 to −2. The implication of this finding is that, as the amplitude of manipulandum motion increases, the model is able to successfully utilise the additional haptic feedback in contrast to the CNS. This suggest that, unlike the model, the CNS only adheres to altered haptic feedback when gain is sufficiently close to zero or ‘natural’ gain. Although we did not assess perception, it may be the case that once participants perceive the manipulandum to be non‐stationary, this may influence the degree of adherence to the stimulus. In support of this, previous research suggests that the effect of a moving haptic stimulus upon sway is amenable to cognitive influences such as prior experience and expectation. When participants are made aware that the object they are touching may move, postural responses can be suppressed (Bryanton et al. 2019; Vérité and Bachta 2021). In the experiment reported here, although participants were not told which gain condition they were about to experience, they were made aware that the manipulandum may move. It is therefore possible that some suppression may have occurred. However, postural stability was considerably worse during gain = +2 compared to the no‐touch condition. This suggests that participants did not fully suppress haptic feedback signals even when detrimental to balance.

Riley et al. (1999) demonstrated that the sway‐reducing effects of light touch can depend upon instruction. They observed that touch was only beneficial if participants were asked to maintain precise fingertip force. This suggests that the observed reduction in sway may be sub‐serving the touch task rather than due to enhanced haptic feedback. This has been called the ‘suprapostural task’ hypothesis. However, Chen and Tsai (2015) found these two explanations were not mutually exclusive. Depending upon the precise instructions (i.e., maintain touch force with high vs. low precision) *and* the nature of the haptic feedback (useful vs. less useful), either the suprapostural task or the sensory mechanism may dominate. What are the consequences for our findings? We instructed participants to maintain a light finger‐thumb pinch grip throughout, so we cannot rule out the contribution of suprapostural effects. However, the instruction was the same for all gain conditions. Moreover, the observed pattern of sway is difficult to explain using the suprapostural task hypothesis; if reduced sway was purely a consequence of attempting to maintain a precision grip, one might expect to see less sway at extreme gains when the grip task presumably becomes more difficult. But sway was in fact greatest at a gain of +2, suggesting this is not the case. Perhaps more importantly, the model we developed explicitly utilised touch as a sensory feedback control signal and successfully replicated the overall pattern of sway observed empirically, without needing to invoke any kind of suprapostural task. Hence, we believe our findings can be primarily interpreted in the context of sensorimotor control of balance being enhanced (or worsened) by altered haptic feedback.

A limitation of our setup was the delay between movement of the trunk and manipulandum. We estimated this to be 60 ms based upon the lag of the cross‐correlation between the motion tracker and manipulandum position signals. Such delay could potentially affect the extent to which the CNS utilises the haptic signal for sway control. However, postural sway occurs at low frequencies (< 0.3 Hz; Figure 4A,B). The haptic perturbation used here involved a continuous modulation of this low‐frequency signal, rather than discrete pulses. Furthermore, this haptic feedback was successful in reducing sway relative to the no‐touch condition. In terms of model performance, the addition of the 60 ms delay had minimal effect upon the simulated data (not shown here). Hence, it seems unlikely that the delay had a significant influence on sensorimotor control. Nevertheless, future work could empirically investigate the effect of different haptic feedback delays to determine if this is the case.

In addition to understanding basic sensorimotor mechanisms of balance, our findings have relevance for the development of robotic balance assistance and human‐robot interaction in general. With an increasingly ageing population, robot assistants are likely to become an increasingly common feature of life. More generally, as robots become more ubiquitous and sophisticated, human–robot interactions will become more common for the population as a whole. As these interactions will include physical contact, in addition to cognitive interaction, it is therefore important to understand the precise nature of haptic feedback afforded by robots and how they may (or may not) benefit human balance. We have demonstrated the principle that humans can utilise artificially altered haptic feedback gain to improve postural control. Although we were unable to demonstrate improvements beyond that afforded by a static object, an important question is whether this would still apply to populations with impaired sensory function and balance, such as the elderly and those with neurological deficits. In these cases, it will be of interest to know whether increasing feedback gain can indeed offer additional benefits.

## Author Contributions

**Raymond F. Reynolds:** conceptualisation, data curation, formal analysis, funding acquisition, investigation, methodology, project administration, resources, software, supervision, validation, visualisation, writing–original draft. **Craig P. Smith:** conceptualisation, data curation, formal analysis, investigation, methodology, software, validation, visualisation. **Lorenz Assländer:** conceptualisation, data curation, formal analysis, methodology, software, supervision, validation, visualisation.

## Conflicts of Interest

The authors declare no conflicts of interest.

### Peer Review

The peer review history for this article is available at https://www.webofscience.com/api/gateway/wos/peer‐review/10.1111/ejn.16670.

## Data Availability

The datasets used and/or analysed during the current study available from the corresponding author on reasonable request.
